# Supplementary material for: Unveiling the urban colonization of the Asian water monitor (Varanus salvator) across its distribution range using citizen science
Source: PeerJ. 2024 Aug 22;12:e17357. doi: 10.7717/peerj.17357 (PMC11344998; doi:10.7717/peerj.17357)
Supplement: Supplemental Information 1 [file peerj-12-17357-s001.pdf]

SP1. Flow diagram showing the sequential steps developed in conducting the revision, including the descriptive results.

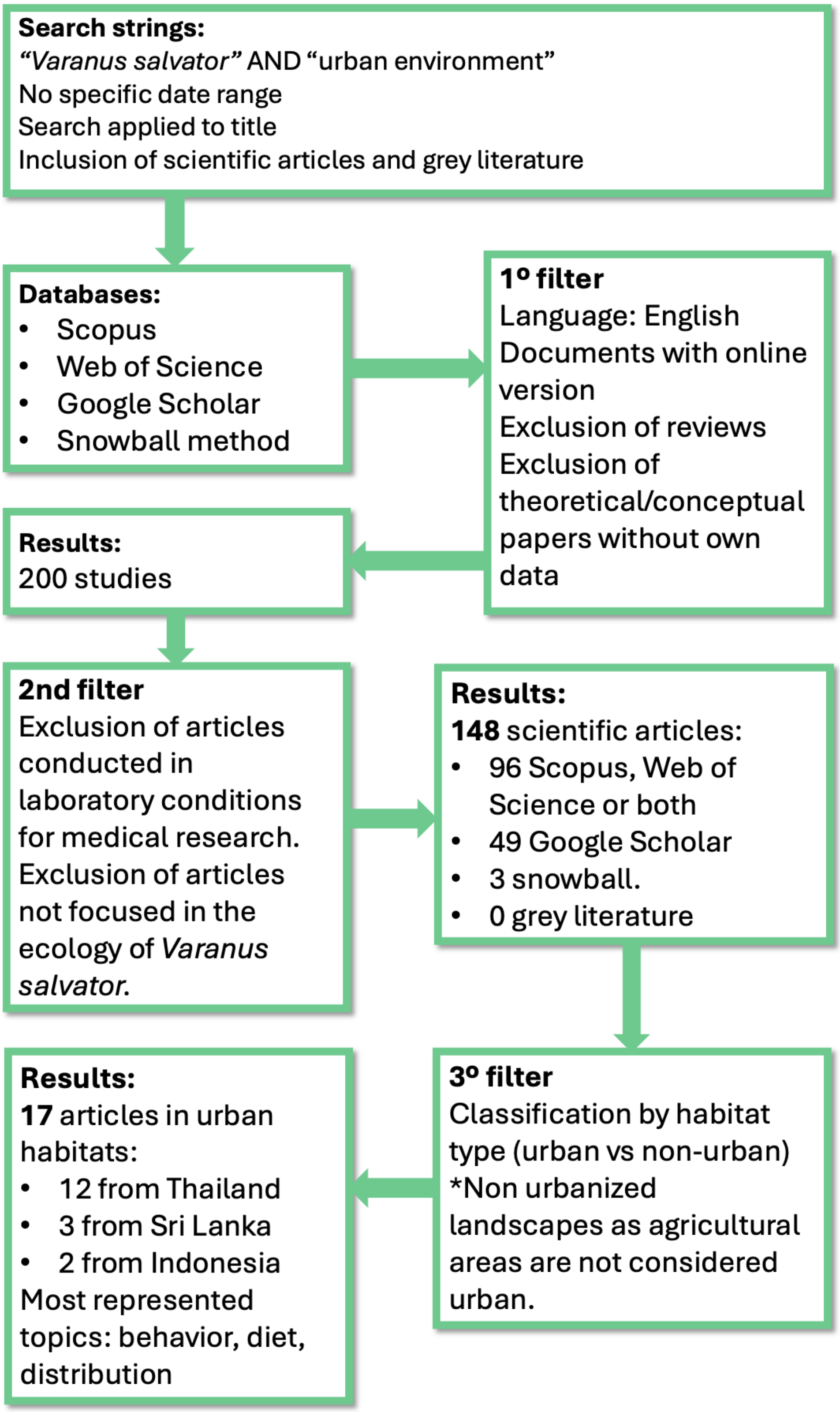

SP2. Criteria used for classifying literature reviewed by topic.

Topic	Description
Behavior	When the study describes aspects of its reproductive, hunting or agonistic behavior.
Diet	When the study describes prey or carrion that the Asian water monitors consume.
Physiology	When the study deals with thermal physiology, hormones or some other aspect regarding its biological functioning.
Distribution	When the study explores how the species is distributed in different regions or ecosystems within their native area.
Habitat selection	When the study focuses on how the species occurs or is absent in a given habitat or microhabitat.
Parasitology	When the study offers information on ecto- and/or endoparasites found in the species.
Conservation	When the study analyzes concerns and threats related to the Asian water monitor conservation

SP3. Summary with all the references retained in reviewed in the literature revision. Urban Ecology works appear first with bold letters. WOS= Web of Science. Other includes Google Scholar.

N	Reference	Year	Country	Source
**1**	**Kulabtong, S., & Mahaprom, R. (2015). Observation on food items of Asian water monitor, Varanus salvator (Laurenti, 1768)(Squamata Varanidae), in urban eco-system, Central Thailand. *Biodiversity Journal*, *6*, 695-698.**	**2015**	**Thailand**	**Other**
**2**	**Stanner, M. (2010). Mammal-like feeding Behavior of Varanus salvator and its Conservational implications. *Biawak*, *4*(4), 128-131.**	**2010**	**Thailand**	**Other**
**3**	**Wongtienchai, P., Lapbenjakul, S., Jangtarwan, K., Areesirisuk, P., Mahaprom, R., Subpayakom, N., ... & Srikulnath, K. (2021). Genetic management of a water monitor lizard (Varanus salvator macromaculatus) population at Bang Kachao Peninsula as a consequence of urbanization with Varanus Farm Kamphaeng Saen as the first captive research establishment. *Journal of Zoological Systematics and Evolutionary Research*, *59*(2), 484-497.**	**2021**	**Thailand**	**WOS/Scopus**
**4**	**Cota, M. (2011). Mating and Intraspecific Behavior of Varanus salvator macromaculatus in an Urban Population. *Biawak*, *5*(1/2), 17-23.**	**2011**	**Thailand**	**Other**
**5**	**Karunarathna, S., Surasinghe, T., Madawala, M., Somaweera, R., & Amarasinghe, A. T. (2017). Ecological and behavioural traits of the Sri Lankan water monitor (Varanus salvator) in an urban landscape of Western Province, Sri Lanka. *Marine and Freshwater Research*, *68*(12), 2242-2252.**	**2017**	**Sri Lanka**	**WOS/Scopus**
**6**	**Cota, M. I. C. H. A. E. L., & Sommerlad, R. A. L. F. (2013). Notes and observations on the fish prey of Varanus salvator macromaculatus (Reptilia: Squamata: Varanidae) in Thailand with a review of the fish prey of the Varanus salvator complex known to date. *Biawak*, *7*(2), 63-70.**	**2013**	**Thailand**	**Other**
**7**	**Cota, M. I. C. H. A. E. L. (2011). Burrows with submerged and waterfilled entrances and nocturnal retirement of Varanus salvator macromaculatus in Thailand. *Biawak*, *5*(3), 44-47.**	**2011**	**Thailand**	**Other**

8	**Takano, A., Kuwata, R., Shimoda, H., Hadi, U. K., Setiyono, A., Agungpriyono, S., & Maeda, K. (2019). Detection and isolation of tick-borne bacteria (Anaplasma spp., Rickettsia spp., and Borrelia spp.) in Amblyomma varanense ticks on lizard (Varanus salvator). *Microbiology and immunology*, *63*(8), 328-333.**	**2019**	**Indonesia**	**Scopus**
9	**Stanner, M. (2012). Varanus salvator swallowing a catfish - Another facet of the life-dinner principle? Hamadryad 36(1), pp. 38-41**	**2012**	**Thailand**	**Scopus**
10	**LAWTON, D., PARLINDUNGAN, D., PRATAMA, A., ASWIN, P., JUNDARA, P., DARMAWAN, R., ... & MATTHEWS, C. E. (1998). Living Among Water Monitors: An Exploratory Study of an Urban Water Monitor (Varanus salvator) Population in Bengkulu, Indonesia. *Biawak*, *12*(1), 42-47.**	**1998**	**Indonesia**	**Other**
11	**BARNES, C. H., TIPPRAPATKUL, W., HRADNANSKY, T., & BARNES, C. (2019). Mobbing Behavior by White-vented Mynas (Acridotheres javanicus) Towards a Southeast Asian Water Monitor (Varanus salvator macromaculatus). *Journal of Varanid Biology and Husbandry*, *13*(1), 46-49.**	**2019**	**Thailand**	**Other**
12	**Hawkeswood, T. J., & Sommung, B. (2016). Observations on the reptile fauna of Lat Krabang Park, Bangkok, Thailand.**	**2016**	**Thailand**	**Other**
13	**Hawkeswood, T. J., & Sommung, B. (2017). Observations on the reptile fauna of Queen Sirikit Park No. 9, Bangkok, Thailand.**	**2017**	**Thailand**	**Other**
14	**Karunarathna, D. M. S. S., Amarasinghe, A. T., & De Vos, A. S. H. A. (2008). Preliminary notes on the Monitor lizards (Family: Varanidae) within the national Zoological Gardens (nZG) dehiwala, Colombo district, sri lanka. *Biawak*, *2*(3), 109-118.**	**2008**	**Sri Lanka**	**Other**
15	**Mahaprom, R., & Kulabtong, S. Observation of feeding habit of the Asian water monitor, Varanus salvator (Laurenti, 1768)(Squamata Varanidae) on a Asian toad, Duttaphrynus melanostictus (Schneider, 1799)(Anura Bufonidae) in Thailand.**	**2018**	**Thailand**	**Other**
16	**Bundhitwongrut, T., Saguensab, S., Thirakhupt, K., and Pauwels, O.S.G. (2008)A case of predation of the Water Monitor Varanus salvator on the Western Snail-eating Turtle Malayemys macrocephala (Reptilia: Varanidae & Bataguridae) in Bangkok**	**2008**	**Thailand**	**Snowball**
17	**Rathnayake, N., Herath, N., Hewamathes, K., and Jayalath, S. (2003). The thermal behaviour, diurnal activity pattern and body temperature of Varanus salvator in Central Sri Lanka. Hamadryad 27, 179–184**	**2003**	**Sri Lanka**	**Snowball**
18	Shine, R., Harlow, P. S., & Keogh, J. S. (1996). Commercial harvesting of giant lizards: the biology of water monitors Varanus salvator in southern Sumatra. *Biological Conservation*, *77*(2-3), 125-134.	1996	Indonesia	Scopus

19	Koch, A., Auliya, M., Schmitz, A., Kuch, U., & Böhme, W. (2007). Morphological studies on the systematics of South East Asian water monitors (Varanus salvator Complex): nominotypic populations and taxonomic overview. *Mertensiella*, *16*(109), e80.	2007	Whole area	Other
20	Shine, R., & Harlow, P. S. (1998). Ecological traits of commercially harvested water monitors, Varanus salvator, in northern Sumatra. *Wildlife Research*, *25*(4), 437-447.	1998	Indonesia	WOS/Scopus
21	De Lisle, H. F. (2007). Observations on Varanus s. salvator in North Sulawesi. *Biawak*, *1*(2), 59-66.	2007	Indonesia	Other
22	Traeholt, C. (1994). The food and feeding behaviour of water monitor, Varanus salvator, in Malaysia. *Malayan Nature Journal (Malaysia)*.	1994	Malaysia	Other
23	Traeholt, C. (1994). Notes on the water monitor Varanus salvator as scavenger. *Malayan Nature Journal (Malaysia)*.	1994	Malaysia	Other
24	Traeholt, C. (1993). Notes of the feeding behaviour of the water monitor, Varanus salvator. *Malayan Nature Journal (Malaysia)*.	1993	Malaysia	Other
25	Ritter, D. A. L. E. (1995). Epaxial muscle function during locomotion in a lizard (Varanus salvator) and the proposal of a key innovation in the vertebrate axial musculoskeletal system. *Journal of experimental biology*, *198*(12), 2477-2490.	1995	XX	WOS/Scopus
26	Uyeda, L. I. N. D. A. (2009). Garbage appeal: relative abundance of water monitor lizards (Varanus salvator) correlates with presence of human food leftovers on Tinjil Island, Indonesia. *Biawak*, *3*(1), 9-17.	2009	Indonesia	Other
27	Gleeson, T. T. (1981). Preferred body temperature, aerobic scope, and activity capacity in the monitor lizard, Varanus salvator. *Physiological Zoology*, *54*(4), 423-429.	1981	XX	WOS
28	Cota, M. I. C. H. A. E. L., Chan-Ard, T. A. N. Y. A., & Makchai, S. U. N. C. H. A. I. (2009). Geographical Distribution and regional variation of Varanus salvator macromaculatus in Thailand. *Biawak*, *3*(4), 134-143.	2009	Thailand	Other
29	Wikramanayake, E. D., & Dryden, G. L. (1993). Thermal Ecology of habitat and microhabitat use by sympatric Varanus bengalensis and V. salvator in Sri Lanka. *Copeia*, 709-714.	1993	Sri Lanka	WOS
30	Akbar, N., Siddiqui, R., Sagathevan, K., Iqbal, M., & Khan, N. A. (2020). Gut bacteria of water monitor lizard (Varanus salvator) are a potential source of antibacterial compound (s). *Antibiotics*, *8*(4), 164.	2019	XX	WOS/Scopus
31	Rahman, K. M., Rakhimov, I. I., & Khan, M. M. H. (2017). Activity budgets and Dietary investigations of Varanus salvator (Reptilia: Varanidae) in Karamjal ecotourism spot of Bangladesh Sundarbans mangrove forest. *Basic and Applied Herpetology*, *31*, 45-56.	2017	Bangladesh	Scopus

32	Welton, L. J., Wood Jr, P. L., Oaks, J. R., Siler, C. D., & Brown, R. M. (2014). Fossil-calibrated phylogeny and historical biogeography of Southeast Asian water monitors (Varanus salvator Complex). *Molecular PhyloGenetics and Evolution*, *74*, 29-37.	2014	XX	WOS/Scopus
33	Gleeson, T. T. (1983). A histochemical and enzymatic study of the muscle fiber types in the water monitor, Varanus salvator. *Journal of Experimental Zoology*, *227*(2), 191-201.	1983	XX	WOS
34	Uyeda, L. I. N. D. A., Iskandar, E. N. T. A. N. G., Purbatrapsila, A. Z. H. A. R. I., Pamungkas, J. O. K. O., Wirsing, A., & Kyes, R. (2014). Water monitor lizard (Varanus salvator) satay: A treatment for skin ailments in Muarabinuangeun and Cisiih, Indonesia. *Biawak*, *8*(1), 35-38.	2014	Indonesia	Other
35	Uyeda, L. I. N. D. A., Iskandar, E. N. T. A. N. G., Wirsing, A. A. R. O. N., & Kyes, R. A. N. D. A. L. L. (2013). Nocturnal activity of Varanus salvator on Tinjil Island, Indonesia. *Biawak*, *7*(1), 25-30.	2013	Indonesia	Other
36	Chia, M. Y., Jeng, C. R., Hsiao, S. H., Lee, A. H., Chen, C. Y., & Pang, V. F. (2009). Entamoeba invadens myositis in a common water monitor lizard (Varanus salvator). *Veterinaryerinary pathology*, *46*(4), 673-676.	2009	XX	WOS/Scopus
37	Mertens, R. (1942). Die familie der warane (Varanidae).	1942	Whole area	Other
38	Borden, R. O. S. S. (2007). Varanus salvator (Asian water monitor) migration. *Biawak*, *1*(2), 84.	2007	Thailand	Other
39	Traeholt, C. A. R. L. (1995). Notes on the burrows of the water monitor lizard, Varanus salvator. *Malayan Nature Journal*, *49*, 103-112.	1995	Malaysia	Other
40	Upton, S. J., & Zien, C. A. (1997). Description of a Giardia varani-like flagellate from a water monitor, Varanus salvator, from Malaysia. *The Journal of Parasitology*, *83*(5), 970-971.	1997	Malaysia	WOS/Scopus
41	Chaiprasertsri, N., Uno, Y., Peyachoknagul, S., Prakhongcheep, O., Baicharoen, S., Charernsuk, S., ... & Srikulnath, K. (2013). Highly species-specific centromeric repetitive DNA sequences in lizards: molecular cytoGenetic characterization of a novel family of satellite DNA sequences isolated from the water monitor lizard (Varanus salvator macromaculatus, Platynota). *Journal of Heredity*, *104*(6), 798-806.	2013	XX	WOS/Scopus
42	Uyeda, L. T., Iskandar, E., Kyes, R. C., & Wirsing, A. J. (2015). Encounter rates, agonistic interactions, and social hierarchy among garbage-feeding water monitor lizards (Varanus salvator bivittatus) on Tinjil Island, Indonesia. *Herpetological Conservation and Biology*, *10*(2), 753-764.	2015	Indonesia	WOS/Scopus

43	Fu, M., Yu, D., Peng, J., Wang, Y., Gao, S., Wang, L., ... & Gao, L. (2011). Isolation and characterization of novel microsatellite markers in Water monitor (Varanus salvator). *Conservation Genetics Resources*, *3*(4), 777-779.	2011	XX	WOS/Scopus
44	Koch, A., & Böhme, W. (2010). Heading east: a new subspecies of Varanus salvator from Obi Island, Maluku Province, Indonesia, with a discussion about the easternmost natural occurrence of Southeast Asian water monitor lizards. *Russian Journal of Herpetology*, *17*(4), 299-309.	2010	Indonesia	Other
45	Tl, C. T. (1997). Activity patterns of free-living water monitor lizards Varanus salvator. *Malayan Nature Journal*, *50*, 301-315.	1997	Malaysia	Other
46	Khadiejah, S., Razak, N., Ward-Fear, G., Shine, R., & Natusch, D. J. (2019). Asian water monitors (Varanus salvator) remain common in Peninsular Malaysia, despite intense harvesting. *Wildlife Research*, *46*(3), 265-275.	2019	Malaysia	WOS/Scopus
47	Mahfud, M., Ernawati, E., Mahmud, N. R. A., Budipitojo, T., & Wijayanto, H. (2020). An immunohistochemical study of endocrine cells in the digestive tract of Varanus salvator (Reptile: Varanidae). *Veterinaryerinary World*, *13*(9), 1737.	2020	XX	WOS/Scopus
48	Mazumder, M. K., Choudhury, A. S., Barbhuiya, R. A., Chakravarty, H., & Barbhuiya, B. (2020). The Ecology, Distribution, status, threats and Conservation of the Common Water Monitor (Varanus salvator) in the Dhaleswari river of Assam, India. *Amphib. Reptile Conserv*, *14*(1), 1-9.	2020	India	WOS/Scopus
49	Amarasinghe, A. A. T., Chathuranga, G., & Karunarathna, D. M. S. S. (2009). Varanus salvator (laurenti, 1768) in rathgama lagoon in Galle district, Sri Lanka. *Biawak*, *3*(3), 81-84.	2009	Sri Lanka	Other
50	Karunarathna, D. M. S. S., Amarasinghe, A. T., & Ekanayake, E. M. K. B. (2008). Observed predation on a suckermouth catfish (Hypostomus plecostomus) by a water monitor (Varanus salvator) in Bellanwila-Attidiya Sanctuary. *Biawak*, *2*(1), 37-39.	2008	Sri Lanka	Other
51	Soopramanien, M., Khan, N. A., Sagathevan, K., & Siddiqui, R. (2021). Gut bacteria of Varanus salvator possess potential antitumour molecules. *International Microbiology*, *24*(1), 47-56.	2021	XX	WOS/Scopus
52	Jeyamogan, S., Khan, N. A., Sagathevan, K., & Siddiqui, R. (2020). Anticancer Properties of Asian Water Monitor Lizard (Varanus salvator), Python (Malayopython reticulatus) and Tortoise (Cuora kamaroma amboinensis). *Anti-Cancer Agents in Medicinal Chemistry (Formerly Current Medicinal Chemistry-Anti-Cancer Agents)*, *20*(13), 1558-1570.	2020	XX	WOS/Scopus

53	Zwart, P., & Harshbarger, J. C. (1972). Hematopoietic neoplasms in lizards: report of a typical case in Hydrosaurus amboinensis and of a probable case in Varanus salvator. *International journal of cancer*, *9*(3), 548-553.	1972	XX	WOS
54	Bhattacharya, S. H. R. E. Y. A., & Koch, A. N. D. R. É. (2018). Effects of traditional beliefs leading to Conservation of water monitor lizards (Varanus salvator) and threatened marshlands in West Bengal, India. *Herpetological Conservation and Biology*, *13*(2), 408-414.	2018	India	WOS/Scopus
55	Doornbos, K., Sumrandee, C., Ruang–Areerate, T., Baimai, V., Trinachartvanit, W., & Ahantarig, A. (2013). Rickettsia sp. closely related to Rickettsia raoultii (Rickettsiales: Rickettsiaceae) in an Amblyomma helvolum (Acarina: Ixodidae) tick from a Varanus salvator (Squamata: Varanidae) in Thailand. *Journal of medical entomology*, *50*(1), 217-220.	2013	Thailand	Other
56	Han, D., & Young, B. A. (2018). Biophysical heterogeneity in the tympanic membrane of the Asian water monitor lizard, Varanus salvator. *Zoomorphology*, *137*(2), 337-348.	2018	XX	WOS/Scopus
57	Lauprasert, K., & THIRAKHUPT, K. (2001). Species diversity, Distribution and proposed status of monitor lizards (family Varanidae) in southern Thailand. *Tropical Natural History*, *1*(1), 39-46.	2001	Thailand	Other
58	Hilditch, T. P., & Paul, H. (1937). The depot fat of Varanus salvator Laur. *Biochemical Journal*, *31*(2), 227.	1937	XX	WOS
59	Jeyamogan, S., Khan, N. A., & Siddiqui, R. (2021). Antitumour Activities of Selected Pure Compounds Identified from the Serum of Crocodylus porosus, Malayopython reticulatus, Varanus salvator and Cuora kamaroma amboinensis. *Asian Pacific Journal of Cancer Prevention*, *22*(S1), 97-106.	2021	XX	Scopus
60	WICKRAMASINgHE, L. M., Kekulandala, L. D. C. B., Peabotuwage, P. K., & Karunarathna, D. M. S. S. (2010). A remarkable feeding Behavior and a new Distribution record of Varanus salvator salvator (Laurenti, 1768) in eastern Sri Lanka. *Biawak*, *4*(3), 93-98.	2010	Sri Lanka	Other
61	Rashid, S. M. A. (2004). *Population Ecology and management of water monitors, Varanus salvator (Laurenti 1768) at Sungei Buloh Wetland Reserve, Singapore* (Doctoral dissertation).	2004	Singapore	Other
62	Choudhury, A. S., & Choudhury, P. A. R. T. H. A. N. K. A. R. (2019). Cruelty to Varanus species of the Barak Valley, Assam, India. *Biawak*, *13*(1), 50-53.	2019	India	Other

63	Han, D., & Young, B. A. (2016). Anatomical basis of dynamic modulation of tympanic tension in the water monitor lizard, Varanus salvator. *The Anatomical Record*, *299*(9), 1270-1280.	2016	XX	WOS/Scopus
64	Rahman, K. M., Rakhimov, I. I., & Khan, M. M. H. (2017). Observation of a Varanus salvator consuming potentially dangerous waste refuse in Karamjal, Bangladesh Sundarbans mangrove forest. *Herpetological Bulletin*, *139*, 33.	2017	Bangladesh	Scopus
65	DUENgKAE, P. R. A. T. E. E. P., & Chuaynkern, Y. O. D. C. H. A. I. Y. (2009). A Road-killed Water Monitor Varanus salvator macromaculatus: Negative Impact from the Forest Route in Khao Yai National park, thailand. *Biawak*, *3*(1), 23Y25.	2009	Thailand	Scopus
66	Myers, G., Webb, T., Corbett, C. R., & Fout, C. (2011). Phacoemulsification for removal of bilateral cataracts in a black water monitor (Varanus salvator macromaculatus). *Journal of Herpetological Medicine and Surgery*, *21*(4), 96-100.	2011	XX	Scopus
67	Rieppel, O., & Labhardt, L. (1979). Mandibular mechanics in Varanus niloticus (Reptilia: Lacertilia). *Herpetologica*, 158-163.	1979	XX	Scopus
68	Wahyuni, S., Jalaluddin, M., & Adnyane, I. K. M. (2015). Studi histokimia sebaran karbohidrat usus biawak air (Varanus salvator). *Acta Veterinaryerinaria Indonesiana*, *3*(2), 77-84.	2015	XX	Other
69	Fitzsimons, J. A. M. E. S., & Thomas, J. (2016). Feeding Behavior of an Asian water monitor Varanus salvator macromaculatus on a Bornean bearded pig Sus barbatus barbatus carcass. *Biawak*, *10*(2), 48-50.	2016	Malaysia	Other
70	Oo, S. S. L., & Bates, P. J. J. (2016). The rediscovery of the common water monitor lizard Varanus salvator (Squamata: Varanidae) in northern Myanmar. *Journal of Threatened Taxa*, *8*(5), 8827-8828.	2016	Myanmar	Scopus
71	Mitchell, G. S., & Gleeson, T. T. (1985). Acid-base balance during lactic acid infusion in the lizard Varanus salvator. *Respiration physiology*, *60*(2), 253-266.	1985	XX	Scopus
72	SRICHAIRAT, N., TAKSINTUM, W., & CHUMNANPUEN, P. (2018). Gross morphological structure of digestive system in water monitor lizard Varanus salvator (Squamata: Varanidae). *Walailak Journal of Science and Technology (WJST)*, *15*(3), 245-253.	2018	XX	Scopus
73	Self, J. T., & Kuntz, R. E. (1966). New Pentastomida. Sambonia parapodum n. sp. from Varanus salvator, and Armillifer agkistrodontis n. sp. from Agkistrodon acutus. *Transactions of the American Microscopical Society*, 256-260.	1966	XX	Scopus
74	cHuA, E. N. G. (2007). Feral Iguana attacks Varanus salvator at sungei Buloh Wetland reserve. *on the Cover: Varanus griseus griseus*, *1*(1), 36.	2007	SIngapore	Other

75	Samarasinghe, D. J., Koch, A., Harikrishnan, S., Manamendra-Arachchi, K., & Chandi, M. (2020). On the Taxonomy and Distribution of Varanus salvator andamanensis Deraniyagala, 1944 (Reptilia: Varanidae), including a redescription of the type specimens and a discussion about its allopatric co-occurrence with V. s. macromaculatus on the Nicobar Islands. *Zootaxa*, *4743*(1), 061-074.	2020	India	WOS
76	Duengkae, P. R. A. T. E. E. P. (2008). Observation of Varanus salvator from Koh Tao Island in the Gulf of Thailand. *Biawak*, *2*(4), 159-161.	2008	Thailand	Other
77	Platt, S. G., Win, M. M., & Rainwater, T. R. (2018). Additional field records provide further resolution of the Distribution of the Water Monitor Varanus salvator (Squamata: Varanidae) in northwestern Myanmar. *Journal of Threatened Taxa*, *10*(10), 12425-12428.	2018	Myanmar	Scopus
78	Burnell, A., Collins, S., & Young, B. A. (2012). The postpulmonary septum of Varanus salvator and its implication for Mosasaurian ventilation and physiology. *Bulletin de la Société Géologique de France*, *183*(2), 159-169.	2012	XX	WOS/Scopus
79	Abdul, J., Hamzah, J., & Wan Abdullah, W. M. (1986). Preliminary study on the growth rate and movement of water monitor lizard (Varanus salvator) at Sungai Tembeling Taman Negara. *The Journal of Wildlife and Parks*, *5*.	1986	Malaysia	Other
80	Traeholt, C. (1995). A radio-telemetric study of the thermoregulation of free living water monitor lizards, Varanus s. salvator. *Journal of Comparative Physiology B*, *165*(2), 125-131.	1995	Malaysia	WOS/Scopus
81	Dryden, G. L., Green, B., Wikramanayake, E. D., & Dryden, K. G. (1992). Energy and water turnover in two tropical varanid lizards, Varanus bengalensis and V. salvator. *Copeia*, 102-107.	1992	Sri Lanka	WOS
82	Ritter, D. A. L. E. (1996). Axial muscle function during lizard locomotion. *Journal of experimental biology*, *199*(11), 2499-2510.	1996	XX	WOS/Scopus
83	Zhang, H., Zhang, S., & Zhang, L. (2011). Two species of the genus Kalicephalus Molin, 1861 (Nematoda, Diaphanocephaloidea) from the water monitor, Varanus salvator (Laurenti, 1768) in Guangdong Province, China. *Acta Parasitologica*, *56*(1), 48-53.	2011	China	WOS/Scopus
84	Villamor, C. I. (1993). Morphometry and Conservation status of water monitor lizard (Varanus salvator) in the Philippines. *Asian International Journal of Life Sciences*.	1993	Philippines	Other
85	Traeholt, C. (1997). Effect of masking the parietal eye on the diurnal activity and body temperature of two sympatric species of monitor lizards, Varanus s. salvator and Varanus b. nebulosus. *Journal of Comparative Physiology B*, *167*(3), 177-184.	1997	XX	WOS/Scopus

86	Uyeda, L. T., Iskandar, E., Kyes, R. C., & Wirsing, A. J. (2012). Proposed research on home ranges and resource use of the water monitor lizard, Varanus salvator. *The Forestry Chronicle*, *88*(5), 542-546.	2012	Indonesia	WOS/Scopus
87	Acharjyo, L. N., Patnaik, M. M., & Patro, K. C. (1970). Treatment of diphyllobothrid tape worm infection in water monitors (Varanus salvator). *Orissa Veterinaryerinary Journal*, *5*(2/3).	1970	XX	Other
88	Mahaprom, R., Duengkae, P., & Chaynkern, Y. (2015). Population density and morphometry analysis for sex determination in Varanus salvator from Bangkachao, Samutprakran Province. *Thai Journal of Forestry*, *34*(3), 109-123.	2015	Thailand	Other
89	Goldthorpe, G., Shepherd, C., Hogg, S., & Leupen, B. (2010). Predation of water monitor lizard (Varanus salvator) by smooth-coated otter (Lutrogale perspicillata) in Peninsular Malaysia. *IUCN Otter Specialists Group Bulletin*, *27*(2), 78-84.	2010	Malaysia	Other
90	Biswas, S. (1981). Some observations on nesting habits and biology of Varanus salvator (Laurenti) of Bhitarkanika Sanctuary, Orissa.	1981	India	Other
91	Liu, W., Li, Q. K., Shih, H. H., & Qiu, Z. Z. (2002). Meristocotyle provitellaria sp. nov.(Digenea: Meristocotylidae) from Varanus salvator in China. *ZOOLOGICAL STUDIES-TAIPEI-*, *41*(3), 283-287.	2002	China	WOS/Scopus
92	Young, B. A., Dumais, J., John, N., Lyons, B., Macduff, A., Most, M., ... & Reiser, P. J. (2016). Functional segregation within the muscles of aquatic propulsion in the Asiatic water monitor (Varanus salvator). *Frontiers in physiology*, *7*, 380.	2016	XX	WOS/Scopus
93	Mulyani, S., Masyitha, D., Wahyuni, S., & Jalaluddin, M. (2015). The anatomical and histological morphology of intestinal water monitor (Varanus salvator). *Jurnal Veterinaryeriner*, *16*(2), 152-158.	2015	XX	Other
94	Fu, M., Yu, D., Wang, Y., & Peng, J. (2011). RAPD analysis for Genetic diversity of Varanus salvator. *Agricultural Science & Technology-Hunan*, *12*(3), 400-404.	2011	whole area	Other
95	Agustin, A. L. D., Koesdarto, S., Lukiswanto, B. S., Suwanti, L. T., Arifin, Z., & Putranto, E. D. (2017). Morphological Identification Nematodes Tanqua tiara Found on Gastric Varanus salvator at East Java. *KnE Life Sciences*, 668-676.	2017	Indonesia	Other
96	Widyaningsih, R. (2020, April). Histological Structure of Varanus Salvator Intestine. In *Proceeding International Conference on Science and Engineering* (Vol. 3, pp. 121-124).	2020	XX	Other
97	Young, B. A. (1988). The subclavian loop of Varanus salvator. *Copeia*, 1029-1034.	1988	XX	WOS
98	Iqbal, M., Zockler, C., & Syroechkovskiy, E. (2013). White-bellied Sea-Eagle'Haliaeetus leucogaster'attempting to prey on water monitor'Varanus salvator'. *Australian Field Ornithology*, *30*(4), 206-209.	2013	Indonesia	Scopus

99	Koch, A., Auliya, M., & Ziegler, T. (2010). Updated checklist of the living monitor lizards of the world (Squamata: Varanidae). *Bonn Zoological Bulletin*, *57*(2), 127-136.	2010	whole area	Other
100	Dwyer, Q., & Pérez, M. (2007). Husbandry and reproduction of the black water monitor, Varanus salvator komaini. Cría y reproducción del monitor negro de agua, Varanus salvator komaini. *Biawak.*, *1*(1), 13-20.	2007	Malaysia	Other
101	Arida, E., Hidayat, A., Mulyadi, M., Maireda, N. L., Subasli, D. R., & Mumpuni, M. (2020). Consumption and Trade of Asian Water Monitor, Varanus salvator as Reliance on Wildlife for Livelihoods among Rural Communities in North Sumatra, Indonesia. *Journal of Tropical Ethnobiology*, *3*(2), 81-92.	2020	Indonesia	Other
102	Lei, J., Booth, D. T., Rusli, M. U., & Zhang, Z. (2020). Spatial Ecology of Asian Water Monitors Adjacent to a Sea Turtle Nesting Beach. *Zoological Science*, *38*(1).	2020	Malaysia	WOS/Scopus
103	Lim, N., Kelt, D. A., Lim, K. K., & Bernard, H. (2020). Vertebrate scavengers control abundance of diarrheal-causing bacteria in tropical plantations.	2020	Malaysia	WOS/Scopus
104	Yang, J. H., & Chan, B. P. L. (2020). Distribution, status, and Ecology of the water monitor (Varanus salvator) on Hainan Island, and the role of folklore in its Conservation. *HERPETOLOGICAL CONSERVATION AND BIOLOGY*, *15*(2), 427-439.	2020	China	WOS/Scopus
105	Kwak, M. L. (2020). A checklist and key to the tick fauna (Acari: Ixodidae, Argasidae) of Pulau Tioman, Malaysia. *Experimental and Applied Acarology*, *81*(1), 51-58.	2020	Malaysia	WOS/Scopus
106	Winnie, F. Y., Siddiqui, R., Sagathevan, K., & Khan, N. A. (2020). Identification of Antibacterial Molecule (s) from Animals Living in Polluted Environments. *Current pharmaceutical biotechnology*, *21*(5), 425-437.	2020	XX	WOS/Scopus
107	Kaenkan, W., Nooma, W., Chelong, I. A., Baimai, V., Trinachartvanit, W., & Ahantarig, A. (2020). Reptile-associated Borrelia spp. in Amblyomma ticks, Thailand. *Ticks and tick-borne diseases*, *11*(1), 101315.	2020	Thailand	WOS/Scopus
108	de Chambrier, A., Brabec, J., Tran, B. T., & Scholz, T. (2019). Revision of Acanthotaenia von Linstow, 1903 (Cestoda: Proteocephalidae), parasites of monitors (Varanus spp.), based on morphological and molecular data. *Parasitology research*, *118*(6), 1761-1783.	2019	Sri Lanka, Malaysia, and Vietnam	WOS/Scopus
109	Jeyamogan, S., Khan, N. A., Sagathevan, K., & Siddiqui, R. (2019). Sera/organ lysates of selected animals living in polluted environments exhibit cytotoxicity against cancer cell lines. *Anti-Cancer Agents in Medicinal Chemistry (Formerly Current Medicinal Chemistry-Anti-Cancer Agents)*, *19*(18), 2251-2268.	2019	XX	WOS/Scopus
110	Yao, Y. T., Du, Y., Fang, M. C., Lin, L. H., & Ji, X. (2019). Developmental stage does not affect resting metabolic rate in the monitor lizard, Varanus salvator. *Animal Biology*, *69*(2), 199-212.	2019	XX	WOS/Scopus

111	Koh, F. X., Panchadcharam, C., Sitam, F. T., & Tay, S. T. (2018). Molecular investigation of Anaplasma spp. in domestic and wildlife animals in Peninsular Malaysia. *Veterinaryerinary Parasitology: Regional Studies and Reports*, *13*, 141-147.	2018	Malaysia	WOS/Scopus
112	Shaney, K. J., Wostl, E., Hamidy, A., Kurniawan, N., Harvey, M. B., & Smith, E. N. (2017). Conservation challenges regarding species status assessments in biogeographically complex regions: examples from overexploited reptiles of Indonesia. *Oryx*, *51*(4), 627-638.	2017	Indonesia	WOS
113	Twining, J. P., Bernard, H., & Ewers, R. M. (2017). Increasing land-use intensity reverses the relative occupancy of two quadrupedal scavengers. *PloS one*, *12*(5), e0177143.	2017	Malaysia	WOS/Scopus
114	F06 Morphological and molecular detection of Blastocystis in wildlife from Tioman Island, Malaysia	2017	Malaysia	WOS
115	Kurniawan, N., Roziah, N., Fauzi, M. A., & Kurnianto, A. S. (2017, November). From little known area to the extinction race: A survey of herpetofauna in Prevab, Kutai National Park (KNP), Indonesia. In *AIP Conference Proceedings* (Vol. 1908, No. 1, p. 020002). AIP Publishing LLC.	2017	Indonesia	WOS/Scopus
116	Gunawardena, S. A. (2016). Artefactual incised wounds due to postmortem predation by the Sri Lankan water monitor (kabaragoya). *Forensic science, Medicine, and pathology*, *12*(3), 324-330.	2016	XX	WOS/Scopus
117	Bucklitsch, Y., Boehme, W., & Koch, A. (2016). Scale morphology and micro-structure of monitor lizards (Squamata: Varanidae: Varanus spp.) and their allies: implications for systematics, Ecology, and Conservation. *Zootaxa*, *4153*(1), 1-192.	2016	XX	WOS
118	Uyeda, L. T., Iskandar, E., Purbatrapsila, A., Pamungkas, J., Wirsing, A., & Kyes, R. C. (2016). The role of traditional beliefs in Conservation of herpetofauna in Banten, Indonesia. *Oryx*, *50*(2), 296-301.	2016	Indonesia	WOS/Scopus
119	Suzuki, D., Fuse, K., Aizu, M., Yoshizawa, S., Tanaka, W., Araya, K., & Praxaysombath, B. (2015). Reptile diversity in food markets in Laos. *Current herpetology*, *34*(2), 112-119.	2015	Laos	WOS/Scopus
120	Du, Y., Lin, L., Yao, Y., Lin, C., & Ji, X. (2014). Body size and reproductive tactics in varanid lizards. *Asian Herpetological Research*, *5*(4), 263-270.	2014	XX	WOS/Scopus
121	Salakij, C., Salakij, J., Prihirunkit, K., Narkkong, N. A., Sanyathitiseree, P., & Kranjanapitukkul, K. (2014). Quantitative and qualitative morphologic, cytochemical, and ultrastructural characteristics of blood cells in captive Asian water monitors. *Veterinaryerinary clinical pathology*, *43*(4), 538-546.	2014	XX	WOS/Scopus

122	Young, B. A., Most, M. G., Dumais, J., John, N., Lyons, B., Macduff, A., & Reiser, P. J. (2014, January). Multiple perspectives of the functional divisions within the swimming muscles of the Asiatic water monitor (Varanus salvator). In *INTEGRATIVE AND COMPARATIVE BIOLOGY* (Vol. 54, pp. E231-E231). JOURNALS DEPT, 2001 EVANS RD, CARY, NC 27513 USA: OXFORD UNIV PRESS INC.	2014	XX	WOS
123	Srikulnath, K., Uno, Y., Nishida, C., & Matsuda, Y. (2013). Karyotype evolution in monitor lizards: cross-species chromosome mapping of cDNA reveals highly conserved synteny and gene order in the Toxicofera clade. *Chromosome Research*, *21*(8), 805-819.	2013	XX	WOS/Scopus
124	Welton, L. J., Siler, C. D., Oaks, J. R., Diesmos, A. C., & Brown, R. M. (2013). Multilocus phylogeny and B ayesian estimates of species boundaries reveal hidden evolutionary relationships and cryptic diversity in S outheast A sian monitor lizards. *Molecular Ecology*, *22*(13), 3495-3510.	2013	XX	WOS/Scopus
125	Kaiser, H., Taylor, D., Heacox, S., Landry, P., Sanchez, C., Ribeiro, A. V., ... & O'Shea, M. (2013). Conservation education in a post-conflict country: five herpetological case studies in Timor-Leste. *Salamandra*, *49*(2), 74-86.	2013	Timor-Leste	WOS/Scopus
126	Koch, A., Ziegler, T., Boehme, W., Arida, E., & Auliya, M. (2013). Pressing problems: Distribution, threats, and Conservation status of the monitor lizards (Varanidae: Varanus spp.) of Southeast Asia and the Indo-Australian Archipelago. *Herpetological Conservation and Biology*, *8*(3), 1-62.	2013	Whole area	WOS/Scopus
127	Welton, L. J., Siler, C. D., Linkem, C. W., Diesmos, A. C., Diesmos, M. L., Sy, E., & Brown, R. M. (2013). Dragons in our midst: Phyloforensics of illegally traded Southeast Asian monitor lizards. *Biological Conservation*, *159*, 7-15.	2013	Philippines	WOS
128	Young, B. A., Dumais, J., Mcmahon, K., & Burnell, A. L. (2012, April). A tale of two tails: Swimming mechanics in Varanus salvator. In *Integrative and Comparative Biology* (Vol. 52, pp. E354-E354). JOURNALS DEPT, 2001 EVANS RD, CARY, NC 27513 USA: OXFORD UNIV PRESS INC.	2012	XX	WOS
129	Nowak, M. (2010). Parasitisation and localisation of ticks [Acari: Ixodida] on exotic reptiles imported into Poland. *Annals of Agricultural and Environmental Medicine*, *17*(2), 237-242.	2010	XX	WOS/Scopus
130	Amin, O. M., Van Ha, N., & Heckmann, R. A. (2008). New and already known acanthocephalans mostly from mammals in Vietnam, with descriptions of two new genera and species in Archiacanthocephala. *Journal of Parasitology*, *94*(1), 194-201.	2008	Vietnam	WOS/Scopus

131	Leong, T. M., & Lim, K. K. (2003). Herpetofaunal records from Fraser's Hill, Peninsular Malaysia, with larval descriptions of Limnonectes nitidus and Theloderma asperum (Amphibia: Ranidae and Rhacophoridae). *Raffles Bulletin of Zoology*, *51*(1), 123-136.	2003	Malaysia	WOS/Scopus
132	Struck, U., Altenbach, A. V., Gaulke, M., & Glaw, F. (2002). Tracing the Diet of the monitor lizard Varanus mabitang by stable isotope analyses (δ 15 N, δ 13 C). *Naturwissenschaften*, *89*(10), 470-473.	2002	XX	WOS
133	Andrews, H. V. (1995). Sexual maturation in Varanus salvator (Laurenti, 1768), with notes on growth and reproductive effort. *Herpetological journal*, *5*(1), 189-194.	1995	XX	WOS/Scopus
134	Mardiastuti, A., MASY'UD, B. U. R. H. A. N. U. D. D. I. N., GINOGA, L. N., SASTRANEGARA, H., & SUTOPO, S. (2021). Wildlife species used as traditional Medicine by local people in Indonesia. *Biodiversitas Journal of Biological Diversity*, *22*(1).	2021	Indonesia	WOS/Scopus
135	Akbar, N., Siddiqui, R., Sagathevan, K., & Khan, N. A. (2020). Gut bacteria of animals living in polluted environments exhibit broad-spectrum antibacterial activities. *International Microbiology*, *23*(4), 511-526.	2020	XX	WOS/Scopus
136	Natusch, D. J., Aust, P. W., Khadiejah, S., Ithnin, H., Isa, A., Zamzuri, C. K., ... & DeNardo, D. F. (2020). Behavioral and corticosterone responses to carbon dioxide exposure in reptiles. *Plos one*, *15*(10), e0240176.	2020	XX	Scopus
137	Rusil, M. U., Chen, G. N., Booth, D. T., & Lei, J. (2020). Diet preference and activity of Asian water monitor at Chagar Hutang turtle sanctuary. *Journal of Sustainability Science and Management*, *15*(6), 68-74.	2020	Malaysia	Scopus
138	Amin, M. H. F. A., Andriyani, A. P., Sari, S. T., Pratiwi, I. A., Suhargo, L., & Irawan, B. First report of Amblyomma sp. collected from Varanus salvator in Baluran National Park Identified by DNA Barcoding.	2019	Indonesia	Scopus
139	Twining, J. P., & koch, A. (2018). Dietary notes and foraging Ecology of south-east Asian water monitors (Varanus salvator) in Sabah, northern Borneo, Malaysia. *Herpetological Bulletin*, *143*, 31.	2018	Malaysia	Scopus
140	Rahman, K. M., Rakhimov, I. I., & Khan, M. M. H. (2017). Public attitudes toward monitor lizards (reptilia: varanidae): A Conservation challenge in the human-dominated ecosystems of Bangladesh. *Annual Research & Review in Biology*, 1-10.	2017	Bangladesh	Scopus
141	Tanalgo, K. C. (2017). Wildlife hunting by indigenous people in a Philippine protected area: a perspective from Mt. Apo National Park, Mindanao Island. *Journal of Threatened Taxa*, *9*(6), 10307-10313.	2017	Philippines	Scopus

142	Mohd Zain, S. N., Farah Haziqah, M. T., Woh, P. Y., Fazly Ann, Z., Vickneshwaran, M., Mohd Khalid, M. K. N., ... & Suresh, K. (2017). Morphological and molecular detection of Blastocystis in wildlife from Tioman Island, Malaysia. *Tropical BioMedicine*, *34*(1), 249-255.	2017	Malaysia	Scopus
143	Buatip, S., Karntanut, W., & Swennen, C. (2013). Nesting period and breeding success of the Little Egret Egretta garzetta in Pattani province, Thailand. *Forktail*, *29*, 120â.	2013	Malaysia	Scopus
144	Shahrudin, S., Jaafar, I. H., Rahim, N. D. A., & Akil, M. A. M. M. (2011). An annotated checklist of the herpetofauna of Beris Valley, Kedah, Malaysia. *Tropical life sciences research*, *22*(1), 13.	2011	Malaysia	Scopus
145	Rawlinson, P. A., Widjoya, A. H. T., Hutchinson, M. N., & Brown, G. W. (1990). The terrestrial vertebrate fauna of the Krakatau Islands, Sunda Strait, 1883-1986. *Philosophical Transactions of the Royal Society of London. B, Biological Sciences*, *328*(1245), 3-28.	1990	Indonesia	Scopus
146	Chatterjee, A., & Bhattacharyya, S. (2015). Distribution and abundance of monitor lizards (Varanus spp.) in human habitations of south west Bengal: People’s tradition of coexisting with wildlife. *African Journal of Science and Research*, *3*(7), 1-7.	2015	India	Other
147	Andrews, H. V., & Gaulke, M. (1990). Observations on the reproductive biology and growth of the water monitor (Varanus salvator) at the Madras Crocodile Bank. *Hamadryad*, *15*(1), 1-5.	1990	India	Snowball
148	Karunarathna, D. M. S. S., Surasinghe, T. D., De Silva, M. C., Madawala, M. B., Gabadage, D. E., & Botejue, W. M. S. (2015). Dietary habits of Varanus salvator salvator in Sri Lanka with a new record of predation on an introduced clown knifefish, Chitala ornata. *Herpetological Bulletin*, *133*, 23-28.	2015	Sri Lanka	Scopus
